# Extra Supplement.—The Nursing Mirror

**Published:** 1888-12-15

**Authors:** 


The Nursing Mirror.

All Contributions for this Supplement should be addressed "The Nursing Mirror," care of The Editor, 38, Old Jewry, E.C.

### En Passant.

PROVINCIAL ITEMS. A course of lectures on nursing, to which ladies are admitted, has been commenced at Worcester Infirmary.—Thirty-five nurses have been trained, examined, and certificated at the Glasgow Maternity Hospital during the last year.—The matron and head nurse of St. Mary's Cottage Hospital, Hampshire, have both held their present posts for sixteen years.—The *Tablet* says that the sisters of St. Vincent de Paul, who nurse the Cork Children's Hospital, are angels, nuns, nurses, and counsellors all in one.—The increase in the staff of nurses of Guest Hospital, Dudley, by the adoption of a system of training probationers, has been found a great success.—The new nurses' quarters at Hereford Infirmary are now in use.

CONTINENTAL HOSPITALS — Mrs. Brewer's last article in the "Sunday at Home" takes us to Buda-Pesth and Milan, and gives us glimpses of the hospitals in these two towns. The size of these foreign hospitals seems enormous. That at Milan holds 4,000 beds, and has a staff of 250 women nurses, 112 men, and 60 sisters. Four to five thousand out-patients attend daily. The wards are enormous, running the height of the hospital, and down the middle of the larger ones a double row of beds is arranged. The nursing arrangements are in the charge of the Sisterhood of St. Vincent de Paul, and seem to be excellent. Each sister receives board, lodging, and uniform, and 1s. 3d. a day; the nurses receive rather less, but can always rely on a pension if they work long and faithfully at their posts. The Elizabeth Hospital at Buda-Pesth is nursed by the Red Cross Sisters, and twelve probationers are received every six months for training. At the end of two years, if they pass a satisfactory examination, they receive the badge of the Red Cross in the shape of a brooch.

NEW RESTAURANT FOR WOMEN.—Nurses, in common with the rest of their sex, must have experienced difficulty, when out for the day, in finding a nice place to lunch. Ordinary restaurants are always crowded with men, and very expensive; the Aerated Bread Company supply good food, and are cheap; but when a nurse has breakfasted at half-past six, by the time noon arrives she needs a substantial meal. This want has been supplied by that excellent society, the Young Women's Christian Association, who have started a restaurant for women only at 101, Mortimer Street, within two minutes' walk of Oxford Circus. The charges are most moderate, and a large plateful of roast mutton and potatoes can be had for 6d. If the potatoes are rather cold, and you have to wait on yourself, fetch your own knife and fork, &c., remember the price! You can't expect degrading luxury for sixpence. Tea, coffee, and cocoa at a penny a cup are delicious, and a thick slice of bread and jam is only a ½d. Strangers going to the restaurant for the first time will experience some difficulty in finding out all the arrangements: where you get your checks, at which counter tea is served, and at which pudding. A few plain directions in large print might be hung about with advantage, but the meal once secured can be enjoyed in quiet, and the charges are eminently suited to a nurse's slender purse. A reading-room and home are in connexion with the restaurant, or rather the restaurant is connected with them, and in the home are cubicles which cost from 4s. to 4s. 6d. per week. The home is constantly used by nurses who are members of the Young Women's Christian Association.

LAST WEEK'S CONVERSAZIONE.—The managers of the British Nurses' Association are to be congratulated on the success of the conversazione given at the Grosvenor Gallery last week. The music was good, Corney Grain was amusing, and the gathering was eminently picturesque. The absence of the Princess Christian and the too-crowded state of the rooms were the only drawbacks to the entertainment. Amongst the guests were Mrs. Jeune, the stately Mrs. Stuart-Wortley, and many other well-known philanthropists. A large number of nurses from many different hospitals enjoyed a pleasant evening.

EMIGRATION FOR WOMEN.—Mrs. Blanchard writing on this subject to "Work and Leisure," announces the fact of the winding-up of the British Ladies' Female Emigrant Society. The objects of the society are now obtained by the Colonial Emigration Society, of 6, Adelphi Terrace, so that the older association has wisely amalgamated with its younger sister. Mrs. Blanchard, speaking of the work done, says: "Trained nurses have been very successful, and many we have sent out have been appointed as matrons to small hospitals up the country, where new towns were forming, and as they become more populated their position and salaries will increase in proportion."

BELFAST NURSES' HOME.—The annual meeting of the Belfast Nurses' Home and Training School took place on December 2nd. Thirty-one candidates entered the home this year, of whom nineteen remained as probationers seventeen of these are now in training; two proved unsuitable. Nine nurses left the home, one to go to Newtownards Union Hospital, one to the Thompson Memorial Home, and one to the Belfast Fever Hospital. During the lady superintendent's holiday Mrs. Llewellyn Davies kindly looked after the management. The financial part of the report was satisfactory, and a legacy of £500 from the late Mr. Hugh M'Calmont was acknowledged.

THE POWER OF PARCHMENT.—In the November number of "Work and Leisure" there was an excellent article on the registration of nurses, entitled "What is Training?" The writer very cleverly described an art student who had passed the South Kensington examinations in freehand, perspective, etc., and secured his certificate. Did that make him a Raphael or a Titian? Nay! it did not even make him an artist. And if this be true of other professions, more especially is it so in the case of nursing, where an unselfish character is as necessary as is an eye for colour and form to an artist. A certificate no more makes a nurse than a like parchment makes an artist. In both cases there is certain preliminary drudgery to be gone through, of which the certificate is the sign; but above and beyond this we reach the true trial as to whether a woman is a good nurse or not. We must look from her certificate to her work, as we look to the art student's picture. And how often then will we find inattention and carelessness; a nurse who is full of faults yet keeps to the windy side of the law, and who cannot be denied her certificate, but who yet lowers the tone of hospitals; and is like a miasma amongst private patients, spreading disrepute of her profession. The writer in "Work and Leisure" pleads for absolute truthfulness on all sides to obviate this evil. Let matrons, doctors, and, above all, private patients, give their true estimate of nurses, and not write "satisfactory" in a report from a cowardly fear of finding fault. It is the danger of the present era of nursing that certificate subjects should be considered the sole necessaries in a nurse, and not those virtues of character which can only faithfully and properly support a woman who permanently undertakes the trying work of nursing.

### Grosvenor Gallery Conversazione.

(By One who was Present.)

We were not honoured with an invitation to the conversazione of the British Nurses' Association, which was given at the Grosvenor Gallery, on December 7th, but a nurse who was present has kindly sent us the following account of the proceedings: "I got away in good time, and reached the Grosvenor about half-past nine. From then till ten I was engaged in squeezing into the cloak-room and in squeezing out again. The crowd was dreadful, and aprons and cap strings were crushed and untidy before ever the galleries were reached. At the door stood Mr. Savory and Miss Wood, busily engaged in shaking hands with each guest who arrived, and further on stood Mrs. Fenwick with a train of black velvet wound around her. The galleries looked very pretty and bright, and I turned my attention first to the Pastels, but there were no catalogues to be had, and before I got very far round the room I was so tightly wedged in the crowd it was impossible to go either back or forward. Then I looked round at the guests, and was so struck at the predominance of Barts' uniforms, I could'nt help wondering who was attending to the patients of that hospital. The severe and picturesque garb of Charing Cross was also well represented, and the pretty red uniforms of Great Ormond Street. There were some nice-looking sisters from Middlesex near me, and one or two from Guy's. The St. John the Evangelist nurses were also in great force, while the Black Johns were conspicuous by their absence. I was the only representative of our hospital, and I saw one solitary sister from the London.

It was noticeable that the matrons of many of the hospitals whose nurses were present, did not put in an appearance, for instance, neither Miss Thorold nor Miss Philippa Hicks were present. Of course the matron of Barts was to the fore, and a number of provincial matrons from the smaller hospitals, including Miss Heanley, whose pamphlet on "Cottage Hospitals" is so well known. The Gray sisters in their Netley uniform, which includes a scarlet cape, looked very nice, and one or two were wearing the Red Cross. There is no doubt about the beauty of nursing uniforms in general, as could be gathered from the happy expression of an artist who had got behind the barriers, and was busy sketching the different head-gears. I hope to see his efforts in one of the illustrated papers next week. I put on my best smile and gazed into space when he turned my way, and wondered if he would condescend to draw my modest cap. In the meanwhile some one was singing a beautiful melody, but I only caught a note now and then intermingled with the chance conversation going on around me. It was getting so hot I made a valiant effort and forced my way out of the gallery into the entrance hall, and then having come specially to see the nursing appliances, I demanded where they were to be found, and fought my way towards them. Here I was very much disappointed, for the exhibition was small and contained few novelties, though everything exhibited was nicely prepared and displayed. Charing Cross, Bartholomew's, Royal Free, and Victoria hospitals, were the only four who had contributed anything. It was not a representative show by any means, and I learnt nothing new, though I spent a long time in examining dressings and appliances. Some of the things looked a little out of place in such an assembly; it is bad enough to come across them unexpectedly in the Parkes Museum if you are going round with a party, but in preparing an exhibition for an assembly, which included many people totally unconnected with hospitals save through some nurse friend, a little more discretion might have been used, especially since there was no attempt at a thorough exhibition.

Having done my duty by the business part of the evening's entertainment, and admired some of the effective Pastels to be seen in that gallery, I made my way downstairs to the refreshment room, which was as uncomfortably crowded as the rest of the building. What with the heat and the crush I could scarcely speak for thirst, but it was only after half-an-hour's waiting that a friend managed to secure an ice for me. I began to regret the despised cold supper my fellow-nurses had been enjoying at the hospital, but as I am very fond of Corney Grain I determined to hear him before I departed. Just then I saw him being piloted with difficulty towards the piano, so I had the lucky inspiration to follow close behind him through the lane opened for him, and thus managed to secure an excellent position for seeing and hearing. The silence which was not vouchsafed to the former singers, was intense directly he took his seat; but I was really sorry for anyone having to perform in such an awful atmosphere. Mr. Grain gave a selection from various musical sketches, all well known at St. George's Hall, and he called forth peals of laughter from everyone who had achieved the victory of squeezing within sound of his voice. I saw my artist friend busy with his pencil again, and hoped he wasn't sketching a lot of laughing nurses, for somehow or other a laughing face doesn't look well under a nurse's cap. Mr. Grain kept us just half-an-hour, by which time it was considerably past eleven, and the long evening of standing, after a hard day's work, had quite done me up. I came to the conclusion that in future I would not seek my pleasure at conversaziones if they were so crowded that chairs could not be found. The squeeze into the cloak-room and out again was just as bad in departing as in arriving, but at last I found my way out into the cool night air, and trundled away home in a cab. As to the guests who were present at the galleries, they certainly chiefly consisted of nurses and matrons; and what specially struck me was that whereas nearly all the nurses belonged to London hospitals, the matrons were nearly all provincial. Which ever way I turned I recognised some face I had known during my career as a nurse, and when I recalled names and asked questions I frequently found my friend was now matron or sister in some small infirmary in a county town. There can be no doubt that the British Nurses' Association had got together a very miscellaneous lot of nurses from all sorts of places, and that many old acquaintances were renewed at the Grosvenor Gallery. There was a very small sprinkling of doctors, chiefly those who are on the council of the Association, including Sir E. Sieveking. I heard that Sir Morell Mackenzie was present, but if so, it must have been for the briefest time, for I never caught a glimpse of him. None of our doctors or students were present. Of nurses' friends there was a pretty large collection, many nurses having evidently thought this a good occasion to arrange a meeting with relatives they do not frequently see. On the whole, it is a pity the British Nurses' Association did not limit the tickets, and so secure greater comfort for their guests, though of course they wished to secure as large a meeting as possible."

### PRIVATE NURSING.

Fitzroy House, Fitzroy Square, W.

The Home Hospital, better known as Fitzroy House, has become a recognised public institution of great utility, mainly owing to the excellence of the administration of the Lady Superintendent, Mrs. Bluett. Experience having shown the necessity for providing a private nursing staff of trained and experienced nurses in connexion with this institution, arrangements have now been completed with this object. The terms are, for ordinary medical and surgical cases, £2 2s. per week, but in addition to this weekly fee, a nurse must be paid her travelling expenses and an allowance of 2s. 6d. per week for laundry Nurses cannot undertake a case for more than two months except by special arrangement, and twenty-four hours' notice must be given to the Lady Superintendent of the intended return of a nurse, who may be withdrawn, however, at any time. The nurses are enjoined to hold secret any knowledge they may obtain of the private affairs of the family in which they are engaged, and are to adapt themselves as far as possible to the usages of the household. A hope is expressed that no gratuity will be offered to any nurse, either by the patients or their friends. Anyone engaging a nurse is required to fill up and sign a form which shows all the expenses paid by the engager to the nurse, including laundry money, railway and cab fares, and also certifies to the nurse's conduct during the time of her engagement, "*a candid expression of opinion being specially invited.*" Nothing is said about uniform, but we hope the Fitzroy House nurses, who are sure to be highly efficient and popular, will have a becoming and distinctive uniform, so that the public may readily recognise and have confidence in every member of the private nursing staff.

### Wages and Washing.—III.

(*Continued from page* xxxix.)

THE SISTERS.—Leaving the nurses for a while to ponder their shortness of wages and washing, we wish to consider the worth of the work done by a hospital sister as valued by a hospital committee. A sister is something more than a trained nurse; she is a woman of capability who can manage a staff of servants and keep accounts. She is the head of a department, and, as such, receives higher remuneration than does a nurse; in fact, a sister is generally considered to be quite a rich person in the nursing world. To what these riches amount we now wish to state, and first for the particulars of the chief London hospitals:—

| Name of Hospital. | Annual Remuneration of Sisters. | | | |
|---|---|---|---|---|
| | Wages. | Uniforms. | Board. | Washing. |
| Guy's | £30 | Out and inside uniforms | complete | complete |
| St. Bartholomew's | Per Week, 25/, 27/6, 30/ | dresses | partial | none |
| St. Thomas's | £50 | partial | complete | complete |
| London | £40—£60 | uniform, except aprons | complete | none |
| St. George's | £35 | dresses | complete | limited |
| Middlesex | £30 | partial | complete | none |
| St. Mary's | £30 | dresses | complete | complete |
| Westminster | £26 5/ | complete | complete | limited |
| King's College | £35 to £50 | dresses | complete | none |

Here it will be seen that the wages vary from £26 to £70 a-year, the highest sum being given at Barts only after long service, and out of it the sisters have to provide their uniform (except the dresses), provide partial board, and pay all their washing. As a matter of fact, the pay is better at Guy's, where wages are only £30; but uniform, board, and washing are all fully provided. With special regard to the washing, it will be seen that at four of the above hospitals it is *nil*, at two it is limited, and at three complete.

THE PROVINCES.—Turning to the provinces, we find the following are the particulars given by nine hospitals:—

| Name of Hospital. | Annual Remuneration of Sisters. | | |
|---|---|---|---|
| | Wages. | Washing. | Uniform. |
| Sunderland Infirmary | £8 | All | All |
| Cheltenham General Hospital | £30 | All | None |
| Kent and Canterbury Hospital | £25-£30 | 18 articles | All |
| Gravesend Hospital | £20-£22 | All | All |
| Victoria Hospital, Burnley | £26 | 28 articles | None |
| Nottingham General Hospital | £25-£30 | All | All |
| Halifax Infirmary | £22-£30 | 24 articles | All |
| Leeds General Infirmary | £25-£40 | All | All |
| Sir Patrick Duns Hospital | £36 | 1s. 6d. | None |

CONCLUSIONS.—These figures can be taken as typical of most county infirmaries, and from them we gather that though wages are lower in the provinces, that terrible item of washing is less. We argued that the very least for which a nurse could get her washing done was £5 a-year, and as a sister has to be even more particular in the way of aprons and caps, which are generally more difficult to do up, we can put the cost of a sister's washing at £7 per annum. Taking the average wage of a sister in a London hospital as £42, this is a sixth of her income. Now when a sixth of £42 is required for washing, another sixth for uniform, and another sixth to eke out the partial board, there is left as clear wages for work done only £21; not exactly a large sum for pocket money, for securing a month's holiday, and for saving from for old age. Next week we hope to conclude our remarks about wages and washing.

### Everybody's Opinion.

[*Correspondence on all subjects is invited. No communications can be entertained if the name and address of the correspondent is not given, or unless one side of the paper only be written on.*]

### THE NATIONAL PENSION FUND FOR NURSES.

MISS K. M. HEANLEY writes:—There is a point regarding the Pension Fund for Nurses which has not been much brought forward, namely, its usefulness to hospital matrons, who, while "in work," have a sufficient income to enable them to pay the premiums required for a yearly pension of £30 to £50 a year, who, when their working days are past, would be left in very straitened circumstances, if not penniless. It has been said by one of the critics of this fund, that only those with incomes precluding the need of a pension could afford to pay these premiums. Surely that critic was unaware of the number there must be of the class mentioned. I think governesses who occupy a similar position would have expressed more gratitude to the promoters of the fund than we nurses and matrons have as yet done. I beg to tender my thanks to those who are endeavouring to help us to help ourselves while we may.

### MASSAGE.

AN American correspondent writes: "Perhaps some of your readers may be interested in the following notes on massage from a book we greatly appreciate here, 'Practical Lessons in Nursing,' by C. R. Mills, M.D., Philadelphia":—"No one should set up as a practitioner of massage without having received special instruction. I have known an individual to undertake to administer massage for pay after observing the operation on one or two occasions. Instruction should be received, and care should be taken that it is of the proper kind. A nurse to-night cannot be a masseur or masseuse to-morrow simply by thinking the matter over. Both teaching and preliminary practice are requisite to make a good operator. The amount of time required to thoroughly learn the art depends upon the intelligence, tact, and manual skill of the individual as well as upon the kind of teaching and the amount of practice. It is not absolutely necessary that masseurs should have a comprehensive knowledge of anatomy and physiology, but they should know something of these branches of medical science; they should know the names, positions, and modes of action of the principal muscles and groups of muscles of the body; they should know something of the size and shape or contour of the muscles in order that they may be better able to isolate and treat them; they should know something of the skin and its functions and powers. Dr. Benjamin Lee defines massage as a communication of motion to the tissues of the living human body from an external source for therapeutic purposes; but this definition does not define the term as it is commonly used—that is, under it might be included Swedish movements and motion communicated by means of steam, electricity, or special mechanical agency. The latter forms of massage are sometimes spoken of as mediates, that is, they are motions or movements communicated to the body through some contrivance or machine. Massage, then, in the sense in which I here employ the term, is the manipulation by a definite process of the tissues and organs by a living operator, for the purpose of promoting health or of relieving or curing disease. A certain knack or tact is soon acquired by a good masseur or masseuse. In most cases wrist and hand movement should be employed. Patients should not be mauled or twisted by movement which call into play the muscles of the arms and trunk, when the whole procedure can be better accomplished by the forearms and hands. Wherever it is possible, the operator should learn to use both hands at once. If the left hand, as in many cases, is comparatively useless, efforts should be made to train it especially, using it in preference to the other hand until the two become equally skilful. To be ambidextrous, or either-handed, is a great advantage in a masseur. Should the massage be dry, or should grease or liniments of some kind be used? Differences of opinion about this practical matter prevail among those who have taught and written upon the subject. Weir Mitchell, for example, advises, or at least permits, in some cases, the use of cocoa-oil or vaseline, but says that these make the work less efficient and more difficult. He does not order them unless it is considered advisable to rub into the system some oleaginous material. Busch and some other writers strongly recommend the employment of oil, liniments, or ointments; Murrell as strongly favours the dry method, saying that the less ointment one uses the better, and that vaseline is never admissible. The only, or almost the only, exception he makes is when the patient suffers from some specific disease, when the operator should use some antiseptic preparation for his own safety and protection. My own view is that nothing in the form of oil, grease, or liniment should be employed. A skilful operator will usually do better without anything of this kind. Some attention must be paid to the direction in which massage is carried out, that is, whether from the periphery towards the centre, or from the centre towards the periphery. When no special orders are given, it is proper to begin at the extremities—at the fingers or toes—and proceed up the limbs, finishing with the muscles of the back, chest, and abdomen. It is of vital importance that those who follow massage as a business should keep in good physical condition. If the number of patients is small, a little extra exercise may be taken. On the other hand, too many patients should not be undertaken in a single day. Six or seven patients are as many as can be done justice to by an operator in good physical condition. It requires considerable strength and vigour to be a good masseur or masseuse; but better even than strength and vigour is the ability to use what strength the individual possesses to the best advantage; in other words, skill. The mistake is made by some operators of supposing that vigorous or even violent treatment is required. Patients should not be roughly handled; on the other hand, it is possible for the treatment to be too mild or too slighting in character. A patient should always be thoroughly treated—should be gone over systematically. One part of the body should not be treated with vigour, and the other slighted. An operator, who is not very strong, or who attempts to do too much work in one day, will start out vigorously enough, but before getting through will become fatigued, and in consequence the part of the body which receives attention last will also receive it least. Then follow some remarks about the mental requirements of masseurs, such as, they should have 'common sense.' Inquisitiveness is to be avoided. They, like the doctor, may sometimes be called upon to penetrate the privacy of families, and should, therefore, before all things, learn to mind their own business. A little pleasant talk will do no harm, but too much should be avoided; particularly should they avoid gossiping and boasting of their own powers. The masseur should particularly refrain from talking much in the evening (when the séance is in the evening), and the lights should be turned away from the patient's eyes. The patient should be in a well-ventilated room, say of 70 deg. The chest should be elevated somewhat, so as to promote free and easy respiration. Each limb should be covered after being treated, and the masseur should not unnecessarily expose any part of the patient. The physician should in some cases inform the operator as to the purpose for which massage is being applied, as this will give the masseur more confidence, and make more certainty as to the thorough performance of his work. The treatment should be commenced very gently, gradually increasing in strength as the patient becomes more able to bear it. Care should be taken not to overdo the treatment on the first attempt. The masseur should exercise his judgment as to the amount of vitality and physical strength possessed by the patient, and upon this, in part, the treatment should be based."

### CURIOUS "TEETHING" CUSTOMS.

"It would be a mistake," says Jacobi, "to believe that we are more mediæval than other nations. The measures for relieving the dangers from the cruel attacks by the ambushing teeth upon the unsophisticated baby, prove better than anything else how the maternal (and professional?) minds have been impressed by awe-stricken faith down to the second half of the nineteenth century. According to H. H. Ploss ("Das Kind in Brauche und Sitte der Völker," 1876, Vol. II.) in different parts of Germany, Austria, and Switzerland, they resort to the following measures: A trouser button and dried umbilical cord are kept under the pillow. The tooth of a colt a twelvemonth old is worn around the neck at the time of the increasing moon. The paw of a mole—bitten off—is sewed in (a bag) and worn round the neck, the baby to be licked by dogs. The head of a mouse is used as the paw of a mole. Every female visitor gives the baby a hard boiled egg. The baby is carried to the butcher, who touches the gums with fresh calf's blood. The gums are touched with the tooth of a wolf or with the claw of a crab. The baby is supplied with three morsels from the first meal in the new residence after the wedding; bread from the wedding feast of a newly married couple in good repute; a mass of lind sprouts cut at twelve o'clock on Good Friday. A bone found by accident under the straw mattress. Mother, when first going to church after confinement, kneels on right knee first. A man coming to visit is silently given a coin, touches the gums of the baby three times, and—goes to the tavern." All these customs in cultured Germany, in the nineteenth century!

### LADY GUIDE ASSOCIATION.

An interesting meeting will be held in the gallery of the Royal Society of British Artists, Suffolk Street, Pall Mall, by the kind permission of the President (Mr. Wyke Bayl ss, F.S.A.), on Tuesday, the 18th inst., at half-past eight p.m., when an explanation will be given of the proposed system of working the above institution, which it is hoped will thenceforward take a permanent form. Tickets may be procured from Miss Edith A. Davis, 5, Lauderdale Road, Maida Vale, W., or anyone will be admitted on presenting their visiting card.

### The Nurses' Bookshelf.

[Books for Review should be sent to The Editor, The Lodge, Porchester Square, W.]

**Nursing Notes.***

With the December part "Nursing Notes" concludes the first year of its existence, and publishes an index which shows what excellent work it has done during the last twelve months. Every number has contained some practical article by a well-known medical man, such as Dr. Jamison, on "Burns;" F. R. Humphreys, L.R.C.P., on "Heart Disease,' &c.; and such authorities amongst nurses as Miss Wilson, Miss Twining, and Miss Heanley have also contributed to its pages. The only fault to be found with "Nursing Notes" is the difficulty in obtaining it. Its disposition is too modest and retiring, with the result that another paper which deals with nursing (and whose disposition is quite the contrary), extracts the good things from "Nursing Notes," and unblushingly reprints them entire in its pages. Both Lawson Tait's article on "Sponges" and Dr. Ellis on "How to Stand" have thus been reproduced without acknowledgment, amongst other scissors-and-paste selections in the journal in question.

### Lectures.

A course of thirty lectures on "Elementary Animal Physiology" are now being given by Mr. David Houston, F.L.S., at South Place Institute on Tuesday evenings at eight o'clock. At the same institute on Tuesday evenings at a quarter to seven, Miss Mary E. Knightly is delivering a course of thirty lectures on "Elementary Biology." Fee for single lecture, sixpence.

### Vacancies.

The following vacancies are announced:—

**Sister.**

South Devon Hospital, Plymouth.

**Nurses.**

Stephen Monckton Nurses' Home.

Samaritan Free Hospital.

Kent and Canterbury Hospital; £25.

Great Yarmouth Hospital; £22.

Albany Memorial Hospital; £20.

Rivers Street Home, Bath; £25.

Princess Alice Hospital, Bournemouth.

Rotherham Hospital; £18.

Bradford Infirmary; £25.

Newcastle Infectious Hospital.

Leeds Borough Fever Hospital (Charge); £30.

Nursing Home, Hockerill, Bishop's Stortford; £20.

Southern Hospital, Manchester; £25.

Royal Surrey County Hospital, Guildford (Assistant); £12.

Home Hospital, 16, Fitzroy Square, W.

North London Consumption Hospital; £21.

Hartlepools Hospital; £25.

Victoria Home, Bournemouth; £25.

Cheltenham Nursing Institution.

Southport Nurses' Home.

Swansea Nursing Institution.

Nursing Institution, 96, 97, 98a, Wimpole Street, London, W., Mr. Wilson has several vacancies for thoroughly-trained Nurses.

City Hospital for Infectious Diseases, Newcastle-upon-Tyne; Day and Night Nurses.

Cottage Hospital, Eltham, Kent.

Hampstead Home Hospital and Nursing Institute; Two; £23.

Southport Infirmary and Local Dispensary; £20.

**Midwives.**

Rivers Street Home, Bath.
Children's Hospital, Kingsholm.

**District Nurses.**

Leeds District Nurses' Home.

Edinburgh.

Swansea Nursing Institution.

Ryde, I.W.

**Probationers.**

Whitechapel Infirmary.

Kent Nursing Institution.

Royal Surrey County Hospital, Guildford.

Hampstead Home Hospital and Nursing Institute; £10 10s.

Princess Alice Memorial Hospital, Eastbourne; £16.

### Notes and Queries.

To Correspondents.—1. Questions may be written on post-cards. 2. Advertisements in disguise are inadmissible. 3. In answering a query please quote the number. 4. If a private answer is desired, a stamped addressed envelope must be forwarded.

**Queries.**

(87) *Blind Asylum for a Boy aged Six.*—Mr. James Briscoe, Secretary Infirmary and Dispensary, Bolton, writes: Can you inform me of a Blind Asylum where a poor boy, aged six, could be admitted?

**Answers.**

(87) *Blind Asylum for Boy aged Six.*—Has Mr. Briscoe applied to the authorities of the Bolton Schools and Workshops for the Blind, 2, Tipping Street, Bolton.

*M. Eddy Pailsey and others* are informed that Dr. Longshore Potts' Book, "Discourses to Women on Medical Subjects," is published privately, the authoress residing in America, U.S.

* "Nursing Notes," a Practical Journal for Nurses. Price 2s. 6d. per annum, post free, from Mrs. Nichol, 15, Buckingham Street, Strand.

